# Symptoms of post-traumatic stress disorder in parents of preterm newborns: A systematic review of interventions and prevention strategies

**DOI:** 10.3389/fpsyt.2023.998995

**Published:** 2023-03-08

**Authors:** Gianluigi Laccetta, Maria Di Chiara, Maria Chiara De Nardo, Gianluca Terrin

**Affiliations:** Department of Maternal and Child Health, Policlinico Umberto I, Sapienza University of Rome, Rome, Italy

**Keywords:** post-traumatic stress disorder, parents – psychology, mother, father, newborn, preterm birth, neonatal intensive care unit, interventions

## Abstract

**Background:**

Preterm birth and subsequent NICU admission can be a traumatic experience for parents who may subsequently develop post-traumatic stress (PTS) disorder (PTSD). Given that developmental issues are common among children of parents with PTSD, interventions for prevention and treatment are essential.

**Objective:**

To assess the most effective non-pharmacological interventions to prevent and/or treat PTS symptoms in parents of preterm newborns.

**Methods:**

Systematic review performed in accordance with the PRISMA statements. Eligible articles in English language were searched in MEDLINE, Scopus, and ISI Web of Science databases using the following medical subject headings and terms: “stress disorder, post-traumatic,” “parents,” “mothers,” “fathers,” “infant, newborn,” “intensive care units, neonatal,” and “premature birth.” The terms “preterm birth” and “preterm delivery” were also used. Unpublished data were searched in ClinicalTrials.gov website. All intervention studies published until September 9th, 2022 and including parents of newborns with gestational age at birth (GA_b_) <37 weeks which underwent ≥1 non-pharmaceutical interventions for prevention and/or treatment of PTS symptoms related to preterm birth were included. Subgroup analyses were conducted by type of intervention. The quality assessment was performed according to the criteria from the RoB-2 and the “NIH Quality Assessment Tool for Before-After studies.”

**Results:**

Sixteen thousand six hundred twenty-eight records were identified; finally, 15 articles (1,009 mothers, 44 fathers of infants with GA_b_ ≤ 36^6/7^ weeks) were included for review. A good standard of NICU care (effective as sole intervention: 2/3 studies) and education about PTSD (effective in association with other interventions: 7/8 studies) could be offered to all parents of preterm newborns. The 6-session Treatment Manual is a complex intervention which revealed itself to be effective in one study with low risk of bias. However, the effectiveness of interventions still remains to be definitively established. Interventions could start within 4 weeks after birth and last 2–4 weeks.

**Conclusion:**

There is a wide range of interventions targeting PTS symptoms after preterm birth. However, further studies of good quality are needed to better define the effectiveness of each intervention.

## Introduction

According to the Diagnostic and Statistical Manual of Mental Disorders - Fifth Edition (DSM-5), post-traumatic stress disorder (PTSD) is a psychiatric disease which diagnosis requires exposure to actual or threatened death, serious injury or sexual violence involving the patient himself or a close family member or friend (1). Patients with PTSD experience intrusion symptoms (e.g., recurrent and intrusive distressing memories of the traumatic event, recurrent distressing dreams related to the traumatic event, dissociative reactions in which the individual feels or acts as if the traumatic event was recurring), persistent avoidance of stimuli associated with the traumatic event, negative alterations in cognition and mood associated with the traumatic event such as inability to remember important aspects of the traumatic event, feelings of detachment and destrangement, and marked alterations in arousal and reactivity beginning or worsening after the traumatic event (1). The aforementioned symptoms last for more than 1 month, with subsequent clinically significant distress or functional impairment (1). Furthermore, these symptoms are not attributable to substance misuse or medical conditions (1). Preterm birth entails risk of death or serious injury for the baby, therefore premature delivery and subsequent Neonatal Intensive Care Unit (NICU) admission can be a traumatic experience for parents (2), which may develop depression and anxiety (2, 3). In addition, prevalence rates of PTSD as high as 53 and 33% have been reported among NICU mothers and fathers, respectively (4). The high prevalence of PTSD among parents of preterm newborns can affect parental well-being and even parenting skills: for example, NICU parents can experience problems bonding with their babies because of PTSD (4). The symptoms of this disorder may also affect parental decision to have further children and induce parents to develop negative attitude toward their infants (4).

It has been demonstrated that children of parents with previous diagnosis of PTSD are at higher risk of cognitive delay than other infants; additionally, developmental and emotional issues are common among children of parents with PTSD (4–6). Notably, psychological distress experienced by NICU families persists over time, and a prevalence of post-traumatic stress (PTS) symptoms equal to 27.1% (95% CI, 20.7–33.6%) has been estimated among parents of NICU-admitted babies more than 1 year after birth (7). Thus, family functioning could be negatively impacted for several months, and even 7 years after the birth of a preterm baby (2, 8–10).

Given these premises, interventions to prevent and treat PTS symptoms in parents of preterm babies are essential. A wide variety of interventions targeting these symptoms are available (10–24), however their efficacy remains unclear. A previous attempt of classifying NICU interventions for parental distress, and quantifying their effectiveness, has been done in 2019 by Sabnis et al. (25). However, that systematic review included only experimental studies published before 2017 measuring NICU parental distress and testing interventions during or pertaining to the hospitalization (25). All the studies included in the systematic review by Sabnis et al. (25) reported post-intervention parental anxiety, stress or PTSD; finally, authors concluded that NICU interventions modestly reduced parental distress. However, the effects of each intervention on PTSD were not assessed separately (25).

Our systematic review aims to assess the most effective non-pharmacological interventions to prevent and/or treat PTS symptoms in parents of preterm newborns and, secondly, to evaluate the best timing for administration of these interventions. In the present article, the term PTSD is used when the diagnostic criteria for this psychiatric disorder are satisfied; the presence of PTS symptoms unites both parents with PTSD diagnosis and parents not fulfilling diagnostic criteria but experiencing some symptoms of PTSD after the birth of a premature baby.

## Methods

A systematic review of current available literature was performed in accordance with the Preferred Reporting Items for Systematic Reviews and Meta-Analyses (PRISMA) statements (26).

### Search strategy

A standard systematic search strategy was adopted (27). We conducted electronic searches in MEDLINE, Scopus, and ISI Web of Science databases; only articles in English language were examined. The following medical subject headings and terms (MESH) were used for doing electronic searches: “stress disorder, post-traumatic,” “parents,” “mothers,” “fathers,” “infant, newborn,” “intensive care units, neonatal,” and “premature birth”; the terms “preterm birth” and “preterm delivery” were also used. Finally, we performed manual search of the reference lists of all eligible articles, electronic and manual screening of conference abstracts and documents from relevant organizations (American Psychiatric Association, American Psychological Association, World Health Organization), and search of unpublished data in ClinicalTrials.gov website. The full search strategies for all databases and websites, including any filters and limits used, are described in Supplementary Table S1, in accordance with the PRISMA statements (26).

### Study selection

**Figure 1:**
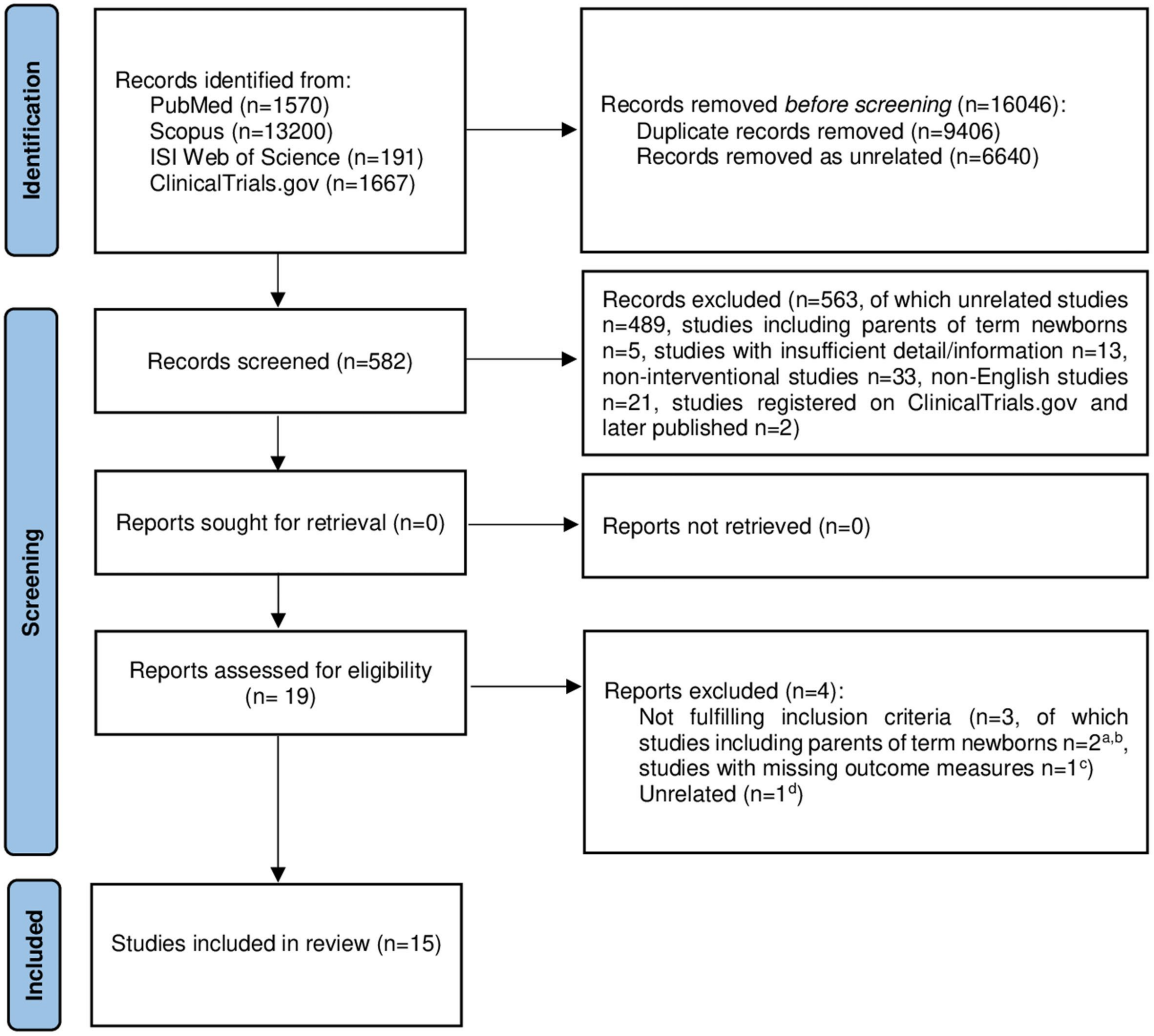
PRISMA 2020 flow diagram showing the selection process of the intervention studies included in our systematic review (28). ^a^Barry et al. (29); ^b^Oyekunle et al. (30); ^c^Jotzo and Poets (31); ^d^Holditch-Davis et al. (32). Adapted with permission from Page et al. (28), licensed under CC-BY 4.0.

We considered eligible all studies fulfilling the following two criteria: (1) Randomized controlled trials (RCTs) and before-after (B-A) studies published until September 9th, 2022; (2) Studies including parents of newborns with gestational age (GA) at birth 22–36 weeks who underwent one or more non-pharmaceutical interventions for prevention and/or treatment of PTS symptoms related to preterm birth. All the authors independently assessed eligibility of related studies for the inclusion according to the previously mentioned criteria; publications with insufficient detail/information, duplicate studies, and articles about interventions for prevention and/or treatment of PTS symptoms related to preterm birth after termination of pregnancy were excluded. In case of different opinions among authors (*n* = 3), consensus was achieved after discussion. A designated form was used to check if studies fulfilled inclusion criteria and to extract necessary information from the selected studies. The study selection form is represented in Supplementary Table S2. The process of literature search and assessment of study eligibility for inclusion is represented in Figure 1 (28).

### Outcome

We evaluated the effectiveness of interventions for prevention and/or treatment of PTS symptoms related to preterm birth in parents of newborns with GA at birth 22–36 weeks. For this purpose, we investigated the severity of PTS symptoms related to preterm birth in parents of newborns with GA at birth 22–36 weeks in terms of mean PTSD score at baseline and after intervention, difference in means of PTSD scores before and after intervention, evaluation of temporal trend of the severity of PTS symptoms through ≥3 measurements (in case the first assessment was performed after intervention was started) or estimate of the longitudinal effect of intervention on PTS symptoms. All the interventions performed in the included studies have been considered. Secondary result was the timing at which each intervention was performed.

### Data extraction

The authors independently extracted data from the selected articles. A specifically designed form summarized the following data for each included article: authorship, publication year, study design, country, number of mothers and fathers enrolled in each study, possible baseline differences among study participants, type of intervention and its timing, questionnaire for PTSD assessment and timing of its administration, details about each intervention and group to which it was administered, effectiveness of interventions (Supplementary Table S2). In case of differences among authors in data extraction, consensus was achieved by discussion. All data extracted from the selected articles were organized into four tables (Supplementary Tables S3–S6) (10–24, 33–39). Thereafter, studies were analyzed in terms of variability in participants, interventions and outcomes. Subgroup analyses were conducted for all interventions performed in the included studies. Given the heterogeneity of patients and methods (interventions, PTSD questionnaires, and timing of interventions and evaluations of PTS symptoms), a meta-analysis of the included studies was not feasible.

### Risk of bias

The quality assessment of included RCTs was performed according to the Cochrane “Risk of Bias assessment tool, 2nd edition” (RoB-2) (40). Within each one of the 5 key domains, the assessment comprised a series of signaling questions for which response options were “yes,” “probably yes,” “probably no,” “no,” and “no information” (40). The risk-of-bias judgments for each domain were “low risk of bias,” “some concerns,” or “high risk of bias”; algorithms mapped responses to signaling questions to a proposed risk-of-bias judgment for each domain (40). The response options for an overall risk-of-bias judgement were the same as for individual domains; the overall risk of bias generally corresponded to the worst risk of bias in any of the domains (40). However, if a study was judged to have “some concerns” about risk of bias for multiple domains, it could be judged as at high risk of overall bias (40).

The quality assessment of included before-after (B-A) studies was performed according to the criteria from the “National Institutes of Health (NIH) Quality Assessment Tool for Before-After (Pre-Post) studies with no control group” available online at https://www.nhlbi.nih.gov/health-topics/study-quality-assessment-tools (41). It included 12 items for which response options were “yes,” “no,” “cannot determine,” “not applicable,” and “not reported” (41). The questions were designed to help reviewers focus on the key concepts for evaluating the internal validity of study (41). Thereafter, the study quality was judged “good,” “fair” or “poor” based on the responses to the signaling questions and the relevance given to key concepts expressed in the signaling questions (41).

When different opinions were found, consensus was reached after discussion.

### Check of methodological quality

The check of the methodological quality of our systematic review was performed in accordance with the statements from the “Assessment of Multiple Systematic Reviews” (AMSTAR) tool (42).

## Results

### Flow of studies

The study selection process is shown in the PRISMA flow diagram (Figure 1) (28). A total of 16,628 records were identified from four databases. After 16,046 references were removed as duplicate (*n* = 9,406) or unrelated (*n* = 6,640) records, 582 studies remained for screening (Figure 1) (28). Each reviewer screened the titles and abstracts of the articles, and 563 studies were excluded as not relevant (unrelated articles, *n* = 489; studies including only parents of term newborns, *n* = 5; studies with insufficient details/information, *n* = 13; non-interventional studies, *n* = 33; articles in languages other than English, *n* = 21; studies registered on ClinicalTrials.gov and later published, *n* = 2). Nineteen references were further evaluated for eligibility: three out of 19 were not considered fulfilling inclusion criteria (studies likely to include only parents of term newborns or parents of both term and preterm infants with no possibility to infer results pertaining only to parents of preterm newborns, *n* = 2; studies with missing outcome measures, *n* = 1) and one reference was excluded as unrelated. Finally, 15 studies met inclusion criteria and were included in our systematic review.

### Characteristics of the included studies

Characteristics of the included studies (10–24) are summarized in Supplementary Table S3 (10–24, 33–39). Three studies have been published by the same group of researchers (10, 21, 22): the first one is a pilot study (21); the second study is a RCT in which the outcome was assessed 4 or 5 weeks after birth (10). The last study is a follow-up study (22) with the same population as the previous one (10) and outcome assessment at 6 months after birth. The studies were performed in 6 countries across North America, Europe and Western Asia: United States (six studies) (10, 12, 16, 21–23), Canada (two studies) (15, 24), Switzerland (two studies) (13, 17), United Kingdom (one study) (11), France (one study) (14), and Iran (three studies) (18–20). Two European RCTs included both mothers and fathers of preterm newborns (2 fathers were included in the study by Barlow et al., and 42 fathers were included in the study by Castel et al.) (11, 14); 11 RCTs and the 2 B-A studies included only mothers of infants with GA at birth up to 36^6/7^ weeks (10, 12, 13, 15–24). Results were provided separately for mothers and fathers only in the study by Castel et al. (14). A total of 1,053 participants (1,009 mothers and 44 fathers) across 15 studies (10–24) were included in our systematic review (patients from the 2 RCTs by Shaw et al. have been counted only once) (10, 22). Details about interventions performed in each study are specified in the next paragraph and summarized in Supplementary Table S4. According to mean (±SD) PTSD score at baseline, 12 studies out of 15 included both patients with PTSD score in the clinical range and parents with PTSD score under the cut-off for PTSD diagnosis (10–15, 17, 18, 21–24). In the studies by Koochaki et al. and Pourmovahed et al., only patients with PTSD score above the cut-off for PTSD diagnosis were included (19, 20). In the study by Holditch-Davis et al., the percentage of patients with PTSD score in clinical range was not specified (16). In most studies, the newborns of enrolled parents have been hospitalized in local NICUs (10–13, 15–24). In the study by Castel et al., newborns were between 28^0/7^ and 35^6/7^ weeks’ GA but it was not clearly specified if all these babies had been admitted to the NICU (14. Interventions were performed during the infant’s NICU stay (12, 18, 21) or after discharge (11, 14); however, in most cases, interventions were started during the infant’s NICU stay and continued even after discharge home (13, 15, 16, 24). In some studies, interventions were started around 1 month after birth (15, 19, 20, 24); in two investigations with the same population, they were started 1–2 weeks after birth (10, 22). Interventions usually lasted 2–4 weeks (10, 12, 18–23); however, in some studies, they lasted more than 4 weeks (13, 16). A single type of questionnaire for PTS symptoms assessment was utilized in each study (10–24): the most used questionnaires were the Perinatal PTSD Questionnaire (PPQ, 7 studies) (13–17, 20, 24) and the Davidson Trauma Scale (DTS, 5 studies) (10, 12, 21–23). However, further tools for PTS symptoms evaluation were also used: Primary Care-PTSD (PC-PTSD, 1 study) (11), Impact of Event Scale-Revised (IES-R, 1 study) (18), and PTSD Symptom Scale (P-SS, 1 study) (19). All five questionnaires were validated for PTSD assessment (10–24) and were based on the diagnostic criteria for PTSD listed in the DSM-4 (11–16, 18–20, 23, 24) or DSM-4-Text Revision (10, 17, 21, 22). When PTSD score was above the specified cut-off for each questionnaire [≥3 for PC-PTSD (34), ≥40 for DTS (12), >6 for PPQ (17), ≥33 for IES-R (18)] or in case one re-experiencing, 3 avoidance and 2 arousal symptoms were contemporarily scored 1 or greater (39), the diagnosis of PTSD was likely. Independently from the diagnosis of PTSD, the scores reported in the aforementioned questionnaires have been used to assess the presence of PTS symptoms and their severity before and after interventions (10–24). With regards to the timing of PTS symptoms assessment, this was performed at baseline (before intervention) in 13 out of 15 studies (10–12, 14, 15, 17–24). Where clearly specified, baseline was considered during the first 4 weeks after birth (10, 18, 20, 22) or after the first 4 weeks of life (12, 15, 17, 19); in two studies, baseline was around 4 weeks after birth (11, 14). In two studies, symptoms evaluation was first performed after the first stage of intervention, 9 or more weeks after birth (13) or at discharge (16). However, in these studies, the temporal trend of the severity of PTS symptoms through multiple assessments (13) or an estimate of the longitudinal effect of intervention on PTS symptoms were provided (16). After the end of interventions, PTS symptoms evaluation was first performed within 2 weeks in most studies (10, 13, 14, 16, 18–22). In 7 out of 15 studies, PTS symptomatology was further assessed a second time (13, 16–19, 22, 23), or even a third time (16), after intervention: in these cases, timing of re-evaluation ranged from 3 weeks after intervention to 12 months’ CA (13, 16–19, 22, 23).

### Interventions

Patients belonging to control groups usually received routinary NICU care and/or discussion of topics related to newborn care (11–18, 20, 24). In both the RCTs by Shaw et al. control group was made up of the same patients who only received education about PTSD, NICU and premature infant’s characteristics and development (10, 22).

Regarding intervention groups, performed interventions were the following ones: (1) Video-recording and joint reviewing of the parent-infant interaction (10, 11, 13, 15, 21, 22, 24); (2) Cognitive restructuring (10, 12, 15, 19, 21–24); (3) Relaxation techniques (10, 12, 15, 19, 21–24); (4) Mindfulness-based stress reduction (18); (5) Facilitation of the father-infant and/or mother-infant interactions, promotion of the parents-infant triadic relationships (10, 14, 15, 21, 22, 24); (6) Auditory-tactile-visual-vestibular stimulation (16); (7) Kangaroo care intervention (16); (8) Expressive writing/trauma narrative (10, 17, 21–23); (9) Non-verbal music (20); (10) Infant redefinition (10, 21–23); (11) Education about the NICU and premature infant’s characteristics and development (10, 12, 14, 21, 22); (12) Education about PTSD (10, 13, 17–19, 21–23). The intervention performed in the study by Feeley et al. was the same as that performed by Zelkowitz et al. (Cues intervention) (15, 24). The interventions performed in the B-A study by Shaw et al. and in the oldest RCT by the same authors were also identical (6-session Treatment Manual) (10, 21). With respect to the latest RCT by Shaw et al., both intervention groups received the 6-session Treatment Manual; however, the intervention group number 2 received 3 further sessions about identification of triggers associated with the development of parental trauma symptoms, and parenting patterns associated with the aspects of the vulnerable child syndrome (22).

In the study by Koochaki et al., both intervention and control group received PTSD counseling, which consisted of education about PTSD, cognitive restructuring, and relaxation techniques: the difference was that control group received an educational package containing these topics, whereas intervention group discussed about the same themes in the PTSD counseling sessions (19).

Details about the interventions performed in the included studies are summarized in Supplementary Table S4 (10–24).

### Effects of interventions

Three RCTs tested the impact of usual NICU care/discussion about topics related to newborn care on PTS symptoms (17, 18, 24). Two out of these studies demonstrated that approach consisting in NICU care and discussion about care was efficacious in prevention and treatment of PTS symptoms in parents of preterm newborns (18, 24).

According to the study by Pourmovahed et al., non-verbal music was also effective for the treatment of PTSD (20); on the contrary, auditory-tactile-visual-vestibular stimulation and kangaroo care intervention were not shown to be efficacious (16).

As concerns combined interventions, education about PTSD, together with one of the following interventions, was demonstrated to be effective in prevention and treatment of PTS symptoms in parents of preterm newborns: (1) Video-recording and joint reviewing of the parent-infant interaction (13); (2) Mindfulness-Based Stress Reduction (18); (3) Education about the NICU and premature infant’s characteristics and development (10); (4) Cognitive restructuring and relaxation techniques (PTSD counseling) (19); (5) Cognitive restructuring, relaxation techniques, expressive writing/trauma narrative and infant redefinition (23). According to Zelkowitz et al., video-recording and joint reviewing of the parent-infant interaction, together with all the following interventions, was effective in prevention and treatment of PTS symptoms in parents of preterm newborns: (1) Cognitive restructuring; (2) Relaxation techniques; (3) Facilitation of the father-infant and/or mother-infant interactions and promotion of the parents-infant triadic relationships (24). Some mixed interventions, which were demonstrated to be effective in reducing PTS symptoms in parents of preterm newborns (13, 17, 19, 23, 24), have been incorporated in the 6-session Treatment Manual (10, 21, 22). Three studies evaluated the effectiveness of this set of interventions in prevention and treatment of PTS symptoms, but results are contrasting (10, 21, 22). If one article did not demonstrate the efficacy of this approach (21), other two studies including a larger number of patients showed that the 6-session Treatment Manual significantly reduced PTS symptoms at brief and long term (10, 22). However, the B-A study by Shaw et al., which failed in demonstrating the effectiveness of this mixed intervention, included only data about a group of 8 mothers whose infants weighed more than 1,000 g at birth (21). Given that both the RCTs proved the effectiveness of the 6-session Treatment Manual on mothers of newborns with birth weight > 600 g, this difference could be relevant in explaining the contrasting results (10, 22). In addition, the 6-session Treatment Manual revealed itself to be more effective than education about the NICU and premature infant’s characteristics and development together with education about PTSD (10, 22).

The following interventions did not lead to a further decrease of the severity of PTS symptoms in relation to usual NICU care and/or discussion about topics related to newborn care: (1) Video-recording and joint reviewing of the parent-infant interaction (11); (2) Auditory-tactile-visual-vestibular stimulation (16; (3) Kangaroo care (16); (4) Cognitive restructuring, relaxation techniques and education about the NICU and premature infant’s characteristics and development (12).

Results from 2 separate RCTs indicated the following interventions as more effective than usual NICU care and/or discussion about topics related to newborn care alone in reducing PTS symptoms in parents of preterm newborns: (1) Non-verbal music (20); (2) Facilitation of the father-infant and/or mother-infant interactions and promotion of the parents-infant triadic relationships together with education about the NICU and premature infant’s characteristics and development (this result is valid for both mothers and fathers separately) (14). In the study by Koochaki et al., PTSD counseling turned out to be more effective than the educational package containing the topics discussed in the PTSD counseling sessions (19).

Expressive writing/trauma narrative, in association with education about PTSD, was demonstrated to be decisive in prevention and treatment of PTSD in parents of preterm newborns in a study with low risk of bias (17). However, in the same study, patients who underwent expressive writing/trauma narrative together with education about PTSD did not achieve a significant decrease in severity of PTS symptoms with respect to patients who only underwent usual NICU care/discussion of topics related to newborn care (17).

Mindfulness-Based Stress Reduction, in association with education about PTSD, was demonstrated to be more efficacious than usual NICU care/discussion of topics related to newborn care in reducing PTS symptoms in parents of preterm newborns (18). However, this finding came from a study with some concerns in risk of bias (18).

Data about the effects of interventions performed in the included studies are summarised in Supplementary Tables S5, S6 (10–24).

### Risk of bias

Data about risk of bias for included RCTs are shown in Supplementary Table S7 (10–20, 22, 24, 40). Risk of bias due to missing outcome data, risk of bias in measurement of the outcome and risk of bias in selection of the reported result were considered low for all included studies (10–20, 22, 24). Overall risk of bias was low for 6 RCTs out of 13 (10, 11, 15–17, 24). In 3 studies, convenience sampling was performed (18–20); in the study by Borghini et al., enough details about the allocation process were not provided (13), thus raising some concerns in risk of bias coming from the randomisation process and overall risk of bias for all 4 studies (13, 18–20). In the RCT by Bernard et al. (12) people delivering the intervention were likely aware of the participants’ assigned intervention, with subsequent concerns in risk of bias due to deviations from intended interventions and overall risk of bias. In 2 further RCTs, there was a high risk of overall bias due to deviations from intended interventions because participants were likely aware of their assigned intervention (14), or no clear information had been given if people delivering the intervention and participants were aware about assigned interventions (22).

Data about risk of bias for included B-A studies are shown in Supplementary Table S8 (21, 23, 41). We considered both B-A studies at high risk of bias because we could not determine if all eligible participants that met the prespecified entry criteria were enrolled, the sample size was not sufficiently large to provide enough confidence in the findings, and outcome measures of interest were not assessed multiple times before and after intervention (21, 23). Furthermore, in the B-A study by Shaw et al., 20 mothers of preterm infants were enrolled but results were given only for 8 of them; similarly, in the study by Simon et al., mothers who completed PTS symptoms assessment were 19 at baseline, 13 at the 6-week follow-up, and only 7 at the six-month follow-up (21, 23).

For most of the included studies, sample size was small, as clearly expressed by Authors (10–14, 16, 19, 21–23). Fathers were not included in most of the selected studies (10, 12, 13, 15–24), and the study by Barlow et al. included only 2 fathers (11).

## Discussion

### Outcomes

This systematic review evaluates, for the first time, the effectiveness of non-pharmacological interventions for selective prevention and treatment of PTS symptoms in parents of preterm newborns. We found that a good standard of NICU care, education about PTSD, and the 6-session Treatment Manual could be suitable for this purpose; these interventions could be administered within 4 weeks after premature birth and NICU admission and could last 2–4 weeks. In their systematic review, Sabnis et al. (25) found that NICU interventions modestly reduce parental distress; however, the effectiveness of each intervention was not selectively assessed on PTS symptoms and studies published after 2017 were not included (25). We think that preterm birth is only the first traumatic event that parents of premature babies have to experience; NICU admission and the course of complications may represent further traumatic events and may contribute to the onset of PTSD in parents of preterm newborns. Our systematic review points out that a wide range of interventions are available for parents of preterm newborns and NICU-admitted infants.

Usual NICU care and discussion about topics related to newborn care turned out to be effective in the RCT by Zelkowitz et al. (24) which also presented a low overall risk of bias. We think that a good standard of care with cooperation among parents, nurses and neonatologists should always be offered to parents of NICU-admitted newborns; a further study with some concerns in risk of bias confirmed this finding (18). This result is in accordance with previous studies demonstrating that a good standard of NICU care with discussion about topics related to newborn care and early involvement of parents was protective against stress in parents of preterm newborns (43, 44).

Education about PTSD is a component of many interventions which turned out to be effective in reducing PTS symptoms in parents of preterm newborns (10, 13, 17–19, 22, 23); thus, we think it could be offered to parents of NICU-admitted infants.

Non-verbal music revealed itself to be effective in reducing PTS symptoms in parents of preterm newborns and it could be offered to parents of NICU-admitted babies. However, only one study with some concerns in risk of bias reached this conclusion (20), thus the effectiveness of this intervention needs further assessment. A previous RCT by Carr et al. (45) has already demonstrated that group music therapy was feasible and effective for treatment of PTSD in adult patients who have not sufficiently responded to cognitive-behavioral therapy (CBT).

As regards expressive writing/trauma narrative, a previous systematic review demonstrated some effectiveness of this treatment in patients with PTSD following childbirth (46), thus contributing to define the already well-known role of this intervention in reducing PTS symptoms. A study by Barry et al., which was performed on mothers of infants who were hospitalized in the NICU for at least 1 week in the previous 14 months, demonstrated that journal writing could reduce PTS symptoms in these patients, thus anticipating the findings by Horsch et al. (29).

Accordingly to the results of our systematic review, a recent article has demonstrated the effectiveness of Mindfulness-Based Stress Reduction in lowering the symptoms of PTSD due to different reasons in adult patients (47). For these reasons, Mindfulness-Based Stress Reduction, in association with education about PTSD, could be offered to parents of preterm newborns after confirmation of its role by means of specifically-designed studies with low risk of bias.

With regard to CBT, both NICE and the International Society for Traumatic Stress Studies recommend its use in adult patients with PTSD (48, 49). A further Cochrane supports the efficacy of trauma-focused CBT in patients with acute PTSD (1–3 months following the traumatic event), even if there is evidence that not all acute PTSD sufferers will benefit from this treatment (50). In accordance with these recommendations, many of the studies included in our systematic review contain principles of trauma-focused CBT in association with 2 or more further interventions (10, 12, 15, 19, 21–24). However, interventions containing CBT were demonstrated to be effective in only 5 studies out of 6 in reducing PTS symptoms in the intervention group (10, 19, 22–24). The B-A study by Shaw et al. (21) failed in demonstrating the effectiveness of an intervention containing principles of CBT in reducing PTS symptoms, but the limited number of mothers included in this investigation may have impacted results. Furthermore, interventions containing CBT revealed themselves to be more effective than interventions performed in the control group in only 2 RCTs out of 5 (10, 12, 15, 22, 24): interestingly, in both these RCTs, intervention was started early after birth (10, 22). Given the contrasting results, further well-designed RCTs about trauma-focused CBT are required (50).

Video-recording and joint reviewing of the parent-infant interaction together with education about PTSD, and video-recording and joint reviewing of the parent–infant interaction associated with cognitive restructuring, relaxation techniques and facilitation of interactions and promotion of parents-infant triadic relationships revealed themselves not to be more effective than usual NICU care/discussion of topics related to newborn care in reducing PTS symptoms in parents of preterm newborns (13, 15, 24).

We think that PTSD assessment could be performed first at 4 weeks after birth or even before (baseline PTSD assessment was performed around 4 weeks after birth or before (10, 11, 14, 18, 20, 22) in half of the studies where the timing of baseline evaluation was clearly expressed (10–20, 22)). Intervention could start early, just after baseline PTSD assessment, in order to prevent the accumulation of unprocessed traumatic memories and facilitate bonding with the baby; in the meantime, recurrent exposure to trauma may further sensitize underlying and dormant disorders (51–55). Intervention could last 2–4 weeks, considered its duration in most of the included studies (10, 12, 18–23). Thereafter, PTSD assessment could be performed within 2 weeks after the end of intervention, as in the most part of included studies (10, 13, 14, 16, 18–22). Given that early intervention could be desirable, it is very likely that it could start with the babies still in the NICU: for this reason, intervention could include a first phase, without direct involvement of the baby, which may take place in a separate room near the NICU. The second phase could entail the direct involvement of the babies and their parents, and it could start just after the babies have left the NICU. Future studies will elucidate definitely the best interventions to be put together in a well-structured program, consisting of the two aforementioned phases, and how these interventions will interact and be effective together.

In contrast with a previous Cochrane (50), a recent review demonstrates that there is insufficient evidence to recommend the introduction of any universal early intervention to all individuals exposed to a traumatic event to prevent PTSD (55, 56). Similarly, another literature review found insufficient evidence to estimate the effectiveness of universal interventions in the primary or secondary prevention of PTSD or PTS symptoms following childbirth (55, 57). However, given the higher prevalence of PTSD in parents of preterm newborns (up to 53% in mothers and 33% in fathers) (4) with respect to general postpartum population (20.7% in mothers, 7.2% in fathers at 1 month post-partum) (58), interventions for PTSD prevention could be offered to all parents of NICU-admitted infants. A further reason for offering interventions for PTSD prevention and treatment to all parents of preterm babies is that parents with PTSD have greater difficulties in co-regulating their infants, thus increasing the risk of their children exhibiting social and emotional problems such as anxiety, aggression, depression and disorganized behavior (59, 60). Given other long-term consequences of parental PTSD on infants [e.g., poorer cognitive outcome (61), sleeping and eating difficulties (62)], and the importance of the first months of life for brain and stress-system development, prevention and early treatment of parental PTSD is even more important (63).

Considered that factors other than preterm birth and NICU admission (e.g., childbirth, NICU environment, baby’s general health condition, newborn’s degree of prematurity, previous traumatic experiences, previous history and current psychiatric diseases, as well as other psychiatric problems like depression and anxiety disorders) may influence the subsequent development of PTSD and its severity, a personalized approach should be even more effective in prevention and treatment of PTSD among parents of preterm newborns. Given the harmfulness of ineffective treatments, which may even retraumatise patients, we feel to emphasize the need of further studies of high quality to assess which category of patients may better benefit from a specific intervention.

### Risk of bias

Given that some studies with concerns in overall risk of bias (12, 13, 18–20) or high risk of overall bias (14, 21–23) have been included, our results need to be confirmed by means of further studies with low risk of bias. If we consider only RCTs with low risk of overall bias, the 6-session Treatment Manual would be the only intervention which turned out to be advantageous in reducing PTS symptoms in comparison with control group (10, 11, 15–17, 24). Even if Shaw et al. have performed this intervention for both preventive and therapeutic aims (10, 22, 23), we ask if such a complex intervention could be appropriate for all patients who have undergone a traumatic event or only for those with PTSD diagnosis. We also would like to know if some components of the 6-session Treatment Manual could be more suitable than others for patients who only have undergone a traumatic event and, in the case, which of these components are better for this purpose. However, only further studies of good quality could give answer to our questions.

Unpublished data represent a potential source of bias we should take into account when interpreting the results of our systematic review. Given the possibility that studies suggesting a beneficial intervention effect or a larger effect size are more likely to be published than data pointing in the opposite direction, our systematic review of the published studies could identify a spurious beneficial effect of some interventions (27). As regards unpublished trials, only two investigations would have been potentially eligible for our systematic review [NCT02736136 (64), NCT01566058 (65)]: in the first case (NCT02736136) (64), a detailed protocol study has already been published (66) but results are not available at present. Concerning the second study (NCT01566058), results have not been published; however, the first PTSD assessment was likely performed after intervention was started and only 2 PTS symptoms measurements were planned (65). In addition, some potentially eligible articles belonging to the gray literature or written in languages other than English might not have been included in our systematic review. On the other hand, risk of bias due to missing outcome data was classified as low for all included RCTs (Supplementary Table S7).

### Strengths and limitations

Preterm birth and NICU-hospitalization may have considerable consequences for parents, including the onset of PTSD; parental PTSD may, in turn, have long-term effects on infants in terms of cognitive outcome and behavior (60–62). We have tried to give an overview over such an important topic by means of a clear search strategy involving multiple databases and even the search of unpublished data. We also selected some studies which were not included in the systematic review by Sabnis et al. (14, 17, 21, 25), and studies which have been published later (18–20, 23). For these reasons, we have included a larger number of studies about interventions for prevention and treatment of PTS symptoms in parents of preterm newborns, and evaluated the effects of each intervention on symptoms. However, our systematic review has some limitations. Given that both parents with PTSD score in clinical range and parents with PTSD score under the cut-off for PTSD diagnosis were included in most studies (10–15, 17, 18, 21–24), our population of interest was quite heterogeneous. In the same way, the variability of interventions, PTSD questionnaires, and timing of interventions and evaluations of PTS symptoms did not allow us to perform a meta-analysis of the included studies. Furthermore, some potentially eligible articles belonging to databases we did not check (e.g., Ovid EMBASE, PsycInfo, Cochrane Library) or to the grey literature might not have been selected for inclusion in the present systematic review. The small sample size (10–14, 16, 19, 21–23) and the low number of studies including both mothers and fathers (11, 14), together with other factors (e.g., low participation rate among eligible patients (15), low percentage of multicenter studies (18), prevalence of Caucasian, highly educated, and affluent patients (12)) limit the possibility to generalize the results of the included studies. In particular, the results of our systematic review are applicable only to Western population, even if psychological support available for parents is variable across health services; thus, further studies involving more ethnicities are needed. The inclusion of only English articles could have contributed to cultural bias, and it may also have introduced language bias considering that studies with positive results are likely published in English (27). For 2 studies, the time in which intervention was started and the time in which it was ended were not clearly specified (11, 16): this could be a limit of both studies, given that time spent on intervention could influence the perceived effectiveness of this activity. In most studies, the presence of PTSD symptoms was assessed through self-reported questionnaires (10, 12–24): on one side, this entails a higher risk of bias due to the tendency of participants to answer in socially desirable ways or to possible wrong interpretation of questions; on the other side, the presence of the researcher may affect answers as well (67). Considered the new structure of some updated questionnaires (PC-PTSD, PPQ, P-SS) (68–70) for PTSD assessment based on DSM-5 criteria, and the absence of an updated version for others (DTS, IES-R), the thresholds used in the included studies probably do not fit the criteria according to the DSM-5. Thus, further studies using questionnaires that fit DSM-5 criteria are needed in order to confirm the real benefit of interventions on PTS symptoms. The absence of a long-term follow-up is a further limitation of most included studies: only 3 RCTs had post-intervention PTSD assessment at 12 (13, 16) or 18 months’ CA (14). Given the high percentage of parents with PTSD symptoms more than a year after their NICU experience (4) and their impact on parenting skills (71, 72), the absence of a long-term follow-up was considered as a limitation for most of the included studies (10–12, 15, 17–24). Finally, two B-A studies without control group were included (21, 23): this means that it was not possible to determine if the reduction in symptoms was due to the intervention itself or would have occurred even in the absence of the treatment through the natural recovery process (23).

## Conclusion

Our systematic review points out that a wide range of interventions to prevent and treat PTS symptoms in parents of preterm newborns is available. We have demonstrated that some of these interventions (good-standard NICU care with involvement of parents and education about PTSD, 6-session Treatment Manual) appear promising. In spite of some favorable evidences from current available literature, the effectiveness of further interventions such as expressive writing/trauma narrative, mindfulness-based stress reduction and non-verbal music, still remains to be definitively established. Interventions could start early after birth, and last 2–4 weeks; they could be offered to all parents of preterm newborns. A personalized approach should be even more effective in prevention and treatment of PTS symptoms in parents of NICU-admitted infants; however, further studies are needed to define which category of patients (e.g., patients with PTSD score above the clinical range and patients with PTSD score under the cut-off for PTSD diagnosis) may better benefit from a specific intervention. Given the consequences of parental PTSD on infants and the harmfulness of ineffective treatments, which may even retraumatise patients, further studies with low risk of bias, longer follow-up and possibility to generalize the results are needed.

## Data availability statement

The original contributions presented in the study are included in the article/Supplementary material, further inquiries can be directed to the corresponding authors.

## Author contributions

GL and MDC had the idea for the article and drafted the work. All the authors performed the literature search and study selection, data extraction, and assessment of risk of bias. GT critically revised and approved the final manuscript, contributed as last authorship. All authors contributed to the article and approved the submitted version.

## Conflict of interest

The authors declare that the research was conducted in the absence of any commercial or financial relationships that could be construed as a potential conflict of interest.

## Publisher’s note

All claims expressed in this article are solely those of the authors and do not necessarily represent those of their affiliated organizations, or those of the publisher, the editors and the reviewers. Any product that may be evaluated in this article, or claim that may be made by its manufacturer, is not guaranteed or endorsed by the publisher.
