# Supplementary material for: Symptoms of post-traumatic stress disorder in parents of preterm newborns: A systematic review of interventions and prevention strategies
Source: Front Psychiatry. 2023 Mar 8;14:998995. doi: 10.3389/fpsyt.2023.998995 (PMC10032332; doi:10.3389/fpsyt.2023.998995)
Supplement: Supplementary file 1 [file Table_1.DOCX]

Table 1. Full search strategies for all databases and websites consulted during the selection process of included studies

| **Medline (https://www.nlm.nih.gov/)** |
| --- |
| ("Stress Disorders, Post-Traumatic"[Mesh]) AND "Parents"[Mesh] |
| ("Stress Disorders, Post-Traumatic"[Mesh]) AND "Infant, Newborn"[Mesh] |
| ("Stress Disorders, Post-Traumatic"[Mesh]) AND "Intensive Care Units, Neonatal"[Mesh] |
| ("Stress Disorders, Post-Traumatic"[Mesh]) AND "Premature Birth"[Mesh] |
| **Scopus (https://www.scopus.com/)** |
| ( ALL ( post AND traumatic AND stress AND disorder ) AND ALL ( neonatal AND intensive AND care AND unit ) ) |
| ( ALL ( post AND traumatic AND stress AND disorder ) AND ALL ( premature AND birth ) ) |
| ( ALL ( post AND traumatic AND stress AND disorder ) AND ALL ( newborn ) AND ALL ( mother ) ) |
| ( ALL ( post AND traumatic AND stress AND disorder ) AND ALL ( newborn ) AND ALL ( father ) ) |
| ( ALL ( post AND traumatic AND stress AND disorder ) AND ALL ( preterm AND birth ) ) |
| ( ALL ( post AND traumatic AND stress AND disorder ) AND ALL ( preterm AND delivery ) ) |
| **ISI Web of Science (https://www.webofscience.com/)** |
| post traumatic stress disorder (All fields) and neonatal intensive care unit (All fields) |
| post traumatic stress disorder (All fields) and premature birth (All fields) |
| post traumatic stress disorder (All fields) and newborn (All fields) and mother (All fields) |
| post traumatic stress disorder (All fields) and newborn (All fields) and father (All fields) |
| post traumatic stress disorder (All fields) and preterm birth (All fields) |
| post traumatic stress disorder (All fields) and preterm delivery (All fields) |
| **ClinicalTrials.gov (https://clinicaltrials.gov/)** |
| Condition or disease: “Post Traumatic Stress Disorder” (all studies) |
| **American Psychiatric Association (https://www.psychiatry.org/)** |
| Search: “post traumatic stress disorder” (anyword, all) |
| **American Psychological Association (https://www.apa.org/)** |
| Search: "post traumatic stress disorder" |
| **World Health Organization (https://www.who.int/)** |
| Search: “post traumatic stress disorder” |
