# Supplementary material for: Symptoms of post-traumatic stress disorder in parents of preterm newborns: A systematic review of interventions and prevention strategies
Source: Front Psychiatry. 2023 Mar 8;14:998995. doi: 10.3389/fpsyt.2023.998995 (PMC10032332; doi:10.3389/fpsyt.2023.998995)
Supplement: Supplementary file 2 [file Table_2.DOCX]

| Authors | Year | Inclusion criteria^§^ | Country | Type of study° | Population* | Intervention(s) | Details about intervention(s) | Timing of the intervention(s) | PTSD assessment |
| --- | --- | --- | --- | --- | --- | --- | --- | --- | --- |
|  |  |  |  |  |  |  |  |  |  |
|  |  |  |  |  |  |  |  |  |  |
|  |  |  |  |  |  |  |  |  |  |
|  |  |  |  |  |  |  |  |  |  |
|  |  |  |  |  |  |  |  |  |  |
|  |  |  |  |  |  |  |  |  |  |
|  |  |  |  |  |  |  |  |  |  |

^§^1. English language; 2. Randomized controlled trial or before-after study published until September 9^th^, 2022; 3. Parents of newborns with gestational age at birth 22-36 weeks who underwent one or more non-pharmaceutical interventions for prevention and/or treatment of post-traumatic stress symptoms related to preterm birth

°Randomized controlled trial (RCT) or before-after (B-A) study

*number of mothers, number of fathers, number of patients in the intervention group(s), number of patients in the control group, statistically significant differences between intervention group and control group at baseline

| Authors | Year | Timing of PTSD assessment | Effect measures^ | Results^ⴕ^ | Effect of intervention(s)^∆^ | Risk of bias^α^ | Note(s) | Reference |
| --- | --- | --- | --- | --- | --- | --- | --- | --- |
|  |  |  |  |  |  |  |  |  |
|  |  |  |  |  |  |  |  |  |
|  |  |  |  |  |  |  |  |  |
|  |  |  |  |  |  |  |  |  |
|  |  |  |  |  |  |  |  |  |
|  |  |  |  |  |  |  |  |  |
|  |  |  |  |  |  |  |  |  |

^Mean (±SD) PTSD score at baseline, mean (±SD) PTSD score during intervention, mean (±SD) PTSD score post-intervention, mean (±SD) PTSD score during follow-up, difference in means of PTSD scores before and after intervention, estimate of the longitudinal effect of intervention on PTSD

^ⴕ^Available PTSD scores, difference in means of PTSD scores before and after intervention, estimate of the longitudinal effect of intervention on PTSD; indicate p-value if statistical analysis has been done

^∆^Intervention favouring intervention group or control group or difference not statistically significant

^α^Randomized controlled trials: bias due to deviations from intended interventions (D), bias in measurement of the outcome (Me), bias due to missing outcome data (Mi), overall risk of bias (O), bias arising from the randomisation process (R), bias in selection of the reported result (S)

Table 2. Study selection form
