# Supplementary material for: Symptoms of post-traumatic stress disorder in parents of preterm newborns: A systematic review of interventions and prevention strategies
Source: Front Psychiatry. 2023 Mar 8;14:998995. doi: 10.3389/fpsyt.2023.998995 (PMC10032332; doi:10.3389/fpsyt.2023.998995)
Supplement: Supplementary file 3 [file Table_3.DOCX]

Table 3. Characteristics of the intervention studies included in our systematic review

| Authors  Year | Type of study^29^ | Country | Population | Infants° | Intervention | PTSD assessment | Timing of the intervention | Timing of PTSD assessment | Effect measures |
| --- | --- | --- | --- | --- | --- | --- | --- | --- | --- |
| Barlow et al.  2016^11^ | RCT | United Kingdom | Mothers n=29, fathers n=2  Intervention group, n=16  Control group, n=15  *The number of mothers’ breastfeeding was higher in the intervention group | Intervention group + control group: ≤ 32 weeks’ GA, BW ≤ 2010 g | Video IG | PC-PTSD^30^ | After discharge, at a time that was convenient to families | T_0_: baseline (17-115 days after birth)  T_1_: within 6 weeks post-intervention | a + c |
| Bernard et al.  2011^12^ | RCT | United States | Mothers n=50, fathers n=0  Intervention group, n=25  Control group, n=25  *The number of Hispanic mothers was higher in the intervention group | Intervention group: 31.3 ± 2.8 weeks’ GA, BW 1659.4 ± 539.3 g  Control group: 32.4 ± 2.6 weeks’ GA, BW 1854.8 ± 595.5 g | CBT | DTS^31^ | 2 weeks during the infant’s NICU stay | T_0_: baseline (intervention group= 44.0 days of hospital stay as mean; control group= 42.0 days of hospital stay as mean)  T_1_: post-intervention (one month following the infant’s discharge) | a + c |
| Borghini et al.  2014^13^ | RCT | Switzerland | Mothers n=55, fathers n=0  Intervention group, n=26  Control group, n=29  *The number of firstborn infants was higher in the control group | Intervention group: 30 ± 2 weeks’ GA, BW 1343 ± 366 g  Control group: 30 ± 2 weeks’ GA, BW 1435 ± 419 g | Three-stage intervention: 1) Joint observation; 2) Videotaped NBAS and semi-structured interview based on the Clinical Interview for Parents of High-Risk Infants; 3) Videotaped mother-infant free play with subsequent IG phase | PPQ^32,33^ | 1^st^ stage: 33 weeks after conception  2^nd^ stage: 42 weeks after conception  3^rd^ stage: 4 months’ CA | T_0_: 42 weeks after conception (> 9 weeks after birth)  T_1_: 4 months’ CA  T_2_: 12 months’ CA | b + c + d |
| Castel et al.  2016^14^ | RCT | France | Mothers n=53, fathers n=42  Intervention group, n=52 (29 mothers, 23 fathers)  Control group, n=43 (24 mothers, 19 fathers)  *The number of mothers with trauma history was higher in the intervention group; the number of mothers hospitalized in Kangaroo Care Unit was higher in the control group | Intervention group: 31.7 ± 2.7 weeks’ GA, BW 1689 ± 469 g  Control group: 32.5 ± 2.0 weeks’ GA, BW 1915 ± 459 g | TRT | PPQ^32,33,§^ | After discharge, up to 18 months’ CA | T_0_: baseline (intervention group= 36.3 days of hospital stay as mean; control group= 28.7 days of hospital stay as mean)  T_1_: post-intervention (18 months’ CA) | e |
| Feeley et al.  2012^15^ | RCT | Canada | Mothers n=96, fathers n=0  Intervention group, n=46  Control group, n=50  *Significance tests for differences in chacteristics of the study population were not conducted | Intervention group: 28.0 ± 2.3 weeks’ GA, BW 982.2 ± 284.1 g  Control group: 27.9 ± 2.2 weeks’ GA, BW 979.5 ± 221.6 g | Cues program (intervention group); Care program (control group) | PPQ^32,33^ | During the infant’s NICU stay, starting from an average of 33 days after birth (SD = 12.4). The first 5 sessions took place in the NICU at a frequency of one or two sessions per week; the last session occurred after discharge | T_0_: baseline (after 4 weeks of NICU stay)  T_1_: post-intervention (6 months’ CA) | a + c |
| Holditch-Davis et al.  2014^16^ | RCT | United States | Mothers n=240, fathers n=0  Intervention group n.1, n=78  Intervention group n.2, n=81  Control group, n=81  *The number of first-time mothers was higher in the intervention group n.1 | Intervention group n.1: 27.0 ± 2.8 weeks’ GA, BW 992.8 ± 329 g  Intervention group n.2: 27.2 ± 2.9 weeks’ GA, BW 1021.7 ± 317 g  Control group: 27.4 ± 3.1 weeks’ GA, BW 1023 ± 343 g | Multisensory ATVV intervention (intervention group n.1); KC using the Ludington-Hoe protocol (intervention group n.2); Attention control intervention (control group) | PPQ^32,33^ | During the infant’s NICU stay, since the infants were no longer critically ill and weighed at least 1000 g, until 2 months’ CA | T_0_: at discharge  T_1_: 2 months’ CA (post-intervention)  T_2_: 6 months’ CA (post-intervention)  T_3_: 12 months’ CA (post-intervention) | f* |
| Horsch et al.  2015^17^ | RCT | Switzerland | Mothers n=65, fathers n=0  Intervention group, n=33  Control group, n=32 | Intervention group: 29.6 ± 2.4 weeks’ GA, BW 1159.2 ± 321.9 g  Control group: 29.3 ± 2.7 weeks’ GA, BW 1059.3 ± 250.2 g | Expressive writing | PPQ^32,33^ | At 3 months’ CA | T_0_: baseline (3 months’ CA)  T_1_: post-intervention (4 months’ CA)  T_2_: post-intervention (6 months’ CA) | a + c + d |
| Izadi et al.  2022^18^ | RCT | Iran | Mothers n=60, fathers n=0  Intervention group, n=30  Control group, n=30 | Intervention group: < 37 weeks’ GA, BW 1944 ± 516.27 g  Control group: < 37 weeks’ GA, BW 2133 ± 283.24 g | MBSR sessions | IES-R^34^ | After the infant’s NICU admission; the intervention group learned MBSR in 3 weeks, 2 sessions a week | T_0_: baseline (after the infant’s NICU admission)  T_1_: immediately after the intervention  T_2_: 1 month after the completion of the intervention | a + c + d |
| Koochaki et al.  2018^19^ | RCT | Iran | Mothers n=81, fathers n=0  Intervention group, n=42  Control group, n=39 | Intervention group + control group: < 37 weeks’ GA, BW < 2500 g | PTSD counseling (intervention group); educational package containing the topics discussed in the PTSD counseling sessions (control group) | P-SS^35^ | The intervention was started ≥ 1 month after NICU hospitalization and lasted 4 weeks | T_0_: baseline (≥ 1 month after NICU hospitalization)  T_1_: immediately after the intervention (4 weeks after T_0_)  T_2_: 3 weeks after the intervention | a + c + d |
| Pourmovahed et al.  2021^20^ | RCT | Iran | Mothers n=45, fathers n=0  Intervention group, n=23  Control group, n=22 | Intervention group + control group: GA and BW not available | Non-verbal music | PPQ^32,33^ | The intervention was started 4 weeks after birth and lasted 2 weeks | T_0_: baseline (during the first 4 weeks after birth)  T_1_: post-intervention (2 to 4 weeks after T_0_) | a + c + e |
| Shaw et al.  2013^21^ | B-A | United States | Mothers n=20, fathers n=0  Intervention group, n=20  Control group, n=0  *Preliminary outcome data is presented on a group of eight mothers who participated in the pilot study | Intervention group: 26-34 weeks’ GA, BW > 1000 g | 6-session Treatment Manual | DTS^31^ | The intervention lasted 3 weeks and was performed during the infant’s NICU stay | T_0_: baseline  T_1_: 1 to 2 weeks following the three-week intervention | a + c + e |
| Shaw et al.  2013^10^ | RCT | United States | Mothers n=98, fathers n=0  Intervention group, n=57  Control group, n=41  *Maternal age was higher in the intervention group; PSS:NICU global index, PSS:NICU infant behavior/appearance subscale, and PSS:NICU alteration in parental role subscale were higher in the intervention group | Intervention group: 30.90 ± 3.00 weeks’ GA, BW > 600 g  Control group: 31.56 ± 2.60 weeks’ GA, BW > 600 g | 6-session Treatment Manual (intervention group); one 45-minute information session on the policy, procedures, and environment of the NICU with education about parenting the premature infant, and referral to the existing parent mentor program for support and coping strategies (control group) | DTS^31^ | The intervention was started 1-2 weeks after birth and lasted 3 to 4 weeks | T_0_: baseline (1-2 weeks after birth)  T_1_: 1 week after the completion of the intervention (4 to 5 weeks after birth) | a + f |
| Shaw et al.  2014^22^ | RCT | United States | Mothers n=95, fathers n=0  Intervention group n.1, n=38  Intervention group n.2, n=19  Control group, n=38  *Maternal age was higher in the intervention group; PSS:NICU global index, PSS:NICU infant behavior/appearance subscale, and PSS:NICU alteration in parental role subscale were higher in the intervention group | Intervention group n.1 + Intervention group n.2: 30.90 ± 3.00 weeks’ GA, BW > 600 g  Control group: 31.56 ± 2.60 weeks’ GA, BW > 600 g | 6-session Treatment Manual (intervention groups n.1 and n.2); 3 further sessions (intervention group n.2) about triggers associated with the development of parental trauma symptoms, and education about parenting patterns; one 45-minute information session on the policy, procedures, and environment of the NICU with education about parenting the premature infant, and referral to the existing parent mentor program for support and coping strategies (control group) | DTS^31^ | The intervention was started 1-2 weeks after birth and lasted 3 to 4 weeks | T_0_: baseline (1-2 weeks after birth)  T_1_: 1 week after the completion of the first 6 session of the intervention or 4-5 weeks after birth  T_2_: 6 months after birth | a + f |
| Simon et al.  2021^23^ | B-A | United States | Mothers n=19 (n=13 at six-week follow-up, n=7 at six-month follow-up), fathers n=0  Intervention group, n=19 (n=13 at six-week follow-up, n=7 at six-month follow-up)  Control group, n=0 | Intervention group: 23-34 weeks’ GA, BW not available | Psychoeducation, cognitive restructuring techniques, progressive muscle relaxation, deep breathing, trauma narrative, education about parenting style | DTS^31^ | The intervention lasted 3 weeks | T_0_: baseline  T_1_: 6 weeks after intervention  T_2_: 6 months after intervention | a + c + d |
| Zelkowitz et al.  2011^24^ | RCT | Canada | Mothers n=98, fathers n=0  Intervention group, n=48  Control group, n=50  *Significance tests for differences in chacteristics of the study population were not conducted | Intervention group: 28.0 ± 2.3 weeks’ GA, BW 992.8 ± 280.7 g  Control group: 27.9 ± 2.2 weeks’ GA, BW 973.4 ± 219.8 g | Cues program (intervention group); Care program (control group) | PPQ^32,33^ | The intervention began on average 33 days (SD = 12) after birth; the first five sessions took place in the NICU, whereas the last session occurred at home 2 to 4 weeks after discharge. The intervention ended at 2-6 weeks’ CA | T_0_: baseline  T_1_: post-intervention (2 to 4 weeks after the intervention ended, or 6 to 8 weeks’ CA) | a + c + e |

^a^Mean PTSD score at baseline; ^b^Mean PTSD score during intervention; ^c^Mean PTSD score at post-intervention assessment; ^d^Mean PSTD score during follow-up; ^e^Difference in means of PTSD scores before and after intervention; ^f^Estimate of the longitudinal effect of intervention on PTSD by linear mixed models.

° Baseline characteristics of infants of enrolled parents; *Linear mixed models also indicated no maternal differences in post-traumatic stress symptoms among the groups at enrollment (data not shown); ^§^In the study by Castel et al., the severity of PTS symptoms was assessed by means of the Perinatal PTSD Scale (the same test as the Perinatal PTSD Questionnaire – PPQ^14,32,33^)

Abbreviations:

ATVV, Auditory-Tactile-Visual-Vestibular; B-A, before-after study; BW, birth weight; CA, corrected age; CBT, Cognitive-behavioral therapy; DTS, Davidson Trauma Scale; GA, gestational age; IES-R, Impact of Event Scale-Revised; IG, Interaction Guidance; KC, Kangaroo Care; MBSR, Mindfulness-based stress reduction; NBAS, Neonatal Behavioural Assessment Scale; NICU, Neonatal Intensive Care Unit; P-SS, PTSD symptom scale; PC-PTSD, Primary Care-PTSD; PPQ, Perinatal PTSD Questionnaire; PPS, Perinatal PTSD Scale; PSS:NICU, Parental Stressor Scale: Neonatal Intensive Care Unit; PTSD, post-traumatic stress disorder; RCT, randomized controlled trial; TRT, Triadic parent-infant Relationship Therapy.
