# Supplementary material for: Symptoms of post-traumatic stress disorder in parents of preterm newborns: A systematic review of interventions and prevention strategies
Source: Front Psychiatry. 2023 Mar 8;14:998995. doi: 10.3389/fpsyt.2023.998995 (PMC10032332; doi:10.3389/fpsyt.2023.998995)
Supplement: Supplementary file 4 [file Table_4.DOCX]

| Authors  Interventions | Barlow et al.^11,a^ | Bernard et al.^12,b^ | Borghini et al.^13,c^ | Castel et al.^14,d^ | Feeley et al.^15,e^ | Holditch-Davis et al.^16,f^ | Horsch et al.^17,g^ | Izadi et al.^18,h^ | Koochaki et al.^19,i^ | Pourmovahed et al.^20,j^ | Shaw et al.^21,k^ | Shaw et al.^10,l^ | Shaw et al.^22,m^ | Simon et al.^23,n^ | Zelkowitz et al.^24,o^ |
| --- | --- | --- | --- | --- | --- | --- | --- | --- | --- | --- | --- | --- | --- | --- | --- |
| Video-recording of the parent-infant interaction, editing of the recording and joint reviewing; joint observation of the parent-infant interaction; education about the infant’s states and how to respond appropriately to the baby’s cues; identification of the infants’ stress reactions and self-regulation capacities^a,c,e,k,l,m,o^ | I_1_ |  | I_1_ |  | I_1_ |  |  |  |  |  | I_1_ | I_1_ | I_1_, I_2_ |  | I_1_ |
| Cognitive restructuring^b,e,i,k,l,m,n,o^ |  | I_1_ |  |  | I_1_ |  |  |  | I_1_, C |  | I_1_ | I_1_ | I_1_, I_2_ | I_1_ | I_1_ |
| Relaxation techniques^b,e,i,k,l,m,n,o^ |  | I_1_ |  |  | I_1_ |  |  |  | I_1_, C |  | I_1_ | I_1_ | I_1_, I_2_ | I_1_ | I_1_ |
| Mindfulness-Based Stress Reduction^h^ |  |  |  |  |  |  |  | I_1_ |  |  |  |  |  |  |  |
| Facilitation of the father-infant and/or mother-infant interactions; promotion of the parents-infant triadic relationships^d,e,k,l,m,o^ |  |  |  | I_1_ | I_1_ |  |  |  |  |  | I_1_ | I_1_ | I_1_, I_2_ |  | I_1_ |
| Auditory-tactile-visual-vestibular stimulation^f^ |  |  |  |  |  | I_1_ |  |  |  |  |  |  |  |  |  |
| Kangaroo Care intervention^f^ |  |  |  |  |  | I_2_ |  |  |  |  |  |  |  |  |  |
| Expressive writing/Trauma narrative^g,k,l,m,n^ |  |  |  |  |  |  | I_1_ |  |  |  | I_1_ | I_1_ | I_1_, I_2_ | I_1_ |  |
| Non-verbal music^j^ |  |  |  |  |  |  |  |  |  | I_1_ |  |  |  |  |  |
| Infant redefinition^k,l,m,n^ |  |  |  |  |  |  |  |  |  |  | I_1_ | I_1_ | I_1_, I_2_ | I_1_ |  |
| Education about the NICU and premature infant’s characteristics and development^b,d,k,l,m^ |  | I_1_ |  | I_1_ |  |  |  |  |  |  | I_1_ | I_1_, C | I_1_, I_2_, C |  |  |
| Education about the definition and signs of stress and PTSD; education about common thoughts and feelings among NICU parents; promotion of relationships between parents; expression of emotional experiences about birth and NICU hospitalization within a supportive environment; education about coping strategies^c,g,h,i,k,l,m,n^ |  |  | I_1_ |  |  |  | I_1_ | I_1_ | I_1_, C |  | I_1_ | I_1_, C | I_1_, I_2_, C | I_1_ |  |
| Usual NICU care only/Discussion of topics related to newborn care^a,b,c,d,e,f,g,h,j,o^ | C | C | C | C | C | C | C | C |  | C |  |  |  |  | C |

Abbreviations: I_1_, intervention group n.1; I_2_, intervention group n.2; C, control group.

Table 4. Details of interventions performed in the included studies.

^a^Video Interaction Guidance (intervention group): video-recording of the parent-infant interaction during play or other aspects of daily caregiving + editing of the recording to select micro-moments of interaction that demonstrate the infant’s contact initiatives and the parents’ response to these signals + joint reviewing of the recordings with the parent; control group: usual care; ^b^Cognitive-behavioral therapy (intervention group): education about the NICU and premature infant’s characteristics + cognitive restructuring to reframe negative thoughts related to NICU hospitalization and identify positive self-statements + relaxation techniques (deep breathing, progressive muscle relaxation); control group: usual care; ^c^Intervention group: joint observation, including the mother, a nurse and a therapist of the infant’s reactions and adjustments to various stimuli and his interaction abilities during a standard care procedure in the NICU; the observation was videotaped + videotaped assessment using the Neonatal Behavioural Assessment Scale (NBAS) of the infants’ stress reactions and self-regulation capacities + semi-structured interview with the parents based on the Clinical Interview for Parents of High-Risk Infants, which allowed mothers to express much of their emotional experiences with their infants at birth and during their subsequent hospitalization + videotaped mother-infant free play and subsequent Interaction Guidance phase; control group: usual care; ^d^Triadic parent-infant Relationship Therapy (TRT) (intervention group): facilitation of the father-infant and mother-infant interactions + education about the infant development + promotion of the parents-infant triadic relationships; control group: usual care; ^e^Cues intervention (intervention group): strategies to reduce anxious feelings (e.g., muscle relaxation, imagery, and replacement of anxious thoughts) + exploration of the mother’s own experience about the infant’s interaction cues + discussion about a DVD on infant interactive behaviors + learning exercises to facilitate mother’s interaction with her infant + videotaping during mother-infant interaction + joint reviewing of the recording. Care intervention (control group): discussion about topics related to newborn care; ^f^Multisensory auditory-tactile-visual-vestibular stimulation (intervention group n.1): stimulation in a gradual progression over 15 minutes, beginning with auditory only (voice), then auditory and tactile (moderate stroking or massage), with visual stimulation (eye-to-eye) added as the infant becomes alert; horizontal rocking is added, and tactile component withdrawn in the final 5 minutes. Kangaroo Care intervention (intervention group n.2): the infant is in skin-to-skin contact in an upright position between the mother’s breasts and the side of the infant not in contact with the mother is covered. Attention control intervention (Control group): discussion about how to select and locate safe equipment needed to care for preterm infants at home; ^g^Expressive writing intervention (intervention group): writing about thoughts and feelings related to preterm birth and hospitalization; control group: usual care; ^h^Mindfulness-Based Stress Reduction (intervention group): the concept of mindfulness, explanations about stress, posttraumatic stress, and psychological stress (session 1); the fluid flow of external and internal stimuli, getting rid of the autopilot mode, feeling the world in the moment despite present thoughts, mindful eating exercise and meditation scan (session 2); recognizing our own body, emotions and stressful reactions, fight-or-flight response and meditation scan (session 3); focusing on being in the present tense, practicing seeing and hearing, focusing on the 5 senses, paying attention to body sounds, breathing and thoughts (simultaneously), response and reaction, difficult situations and attitudes, and conscious walking (session 4); defining stress and the body response to it, more efficient response to stress, meditation in daily life, more energy storage to cope with problems, discussion on previous practices, the beginning of the second stage of mindful body movements (session 5); mindful interactions, thought contents are often unrealistic, ways to take more self-care, doing homework in groups of 3, practicing meditation techniques for an hour, 4-dimensional meditation, identifying pleasant and unpleasant events, and finding ways to have fun events and a 3-min practice (session 6); control group: usual care; ^i^PTSD counseling (intervention group): introduction, discussion about group rules and objectives of group therapy, building relationships between mothers (session 1); asking mothers to discuss the psychological aspects of their feelings and thoughts about the hospitalization of their infants, and emotional release within a supportive environment (session 2); discussion about the definition and signs of stress and explaining stress relief techniques (session 3); evaluation of the effects of cognition and thoughts on stress responses, advising the participants on how to identify their negative self-talks, emphasizing the significance of coping skills and discussing how different individuals deal with various stressful events (session 4); discussion about stressful self-talk, and use self-talk as an effective coping strategy (session 5); problem-solving skills, and description of each mother’s problems (session 6); discussion about alternative solutions and choice of the best one (session 7); discussion about the efficacy of each solution (session 8). Control group (C) received an educational package containing the topics discussed in the PTSD counseling sessions. Both groups received education about routine infant care (not shown); ^j^non-verbal music (intervention group) included the sound of rain, sea, and nature, with slow, gentle, and soothing rhythm. The intervention was performed for 20-30 minutes daily during the evening shift (17-18 pm) next to the infant’s incubator; control group: usual care;  ^k^Intervention group received the following treatment: education about symptoms of post-traumatic stress disorder (PTSD) and common feelings and thoughts of NICU parents + cognitive restructuring + progressive muscle relaxation and deep breathing + trauma narrative in either verbal or written form + infant redefinition + facilitation of mother-infant relationship + education about the infant’s states and how to respond appropriately to the baby’s cues + education about infant development (6-session Treatment Manual); ^l^Intervention group received the 6-session Treatment Manual (previously described). Comparison group participants received education about the policy, procedures, and environment of the NICU; mothers were referred to the existing parent mentor program for support and coping strategies; ^m^6-session Treatment Manual as previously described (intervention groups n.1 and n.2); intervention group n.2 received 3 further sessions about identification of triggers associated with the development of parental trauma symptoms, and parenting patterns associated with the aspects of the vulnerable child syndrome. Comparison group participants received education about the policy, procedures, and environment of the NICU; mothers were referred to the existing parent mentor program for support and coping strategies; ^n^Intervention group received the following treatment: cognitive restructuring + relaxation techniques (progressive muscle relaxation and deep breathing) + education about symptoms of PTSD + trauma narrative + infant redefinition; ^o^Cues intervention (intervention group): strategies to reduce anxious feelings (e.g., muscle relaxation, imagery, and replacement of anxious thoughts) + exploration of the mother’s own experience about the infant’s interaction cues + discussion about a DVD on infant interactive behaviors + learning exercises to facilitate mother’s interaction with her infant + videotaping during mother-infant interaction + joint reviewing of the recording. Care intervention (control group): discussion about topics related to newborn care.
