# Supplementary material for: Symptoms of post-traumatic stress disorder in parents of preterm newborns: A systematic review of interventions and prevention strategies
Source: Front Psychiatry. 2023 Mar 8;14:998995. doi: 10.3389/fpsyt.2023.998995 (PMC10032332; doi:10.3389/fpsyt.2023.998995)
Supplement: Supplementary file 5 [file Table_5.DOCX]

Table 5. Results of the included studies based on the effect measures selected for the present systematic review. To be included, studies must contain one or more of the following data set with evaluation of statistical significance, for at least one intervention group: 1) mean PTSD score at baseline and after intervention; 2) difference in means of PTSD scores before and after intervention; 3) estimate of the longitudinal effect of intervention on PTSD

|  | Mean ± SD PTSD score at baseline^§^ | Mean ± SD PTSD score after intervention | Difference in means of PTSD scores before and after intervention | Estimate of the longitudinal effect of intervention on PTSD by general linear models |
| --- | --- | --- | --- | --- |
| Barlow et al.^11^ | I_1_: 1.25 ± 1.48; C: 1.40 ± 1.45 | I_1_: 1.00 ± 1.37; C 1.27 ± 1.16 |  |  |
| Bernard et al.^12^ | I_1_: 32.6 ± 24.7; C: 39.9 ± 25.6 | I_1_: 26.5 (4.7)*; C: 34.6 (4.7)* |  |  |
| Borghini et al.^13^ | I_1_: 4.62 ± 3.54; C: 3.55 ± 2.88° | I_1_: 3.31 ± 3.38; C: 3.59 ± 3.31 (1^st^ assessment)  I_1_: 2.69 ± 2.58; C: 3.17 ± 2.66 (2^nd^ assessment) |  |  |
| Castel et al.^14^ | I_1_: 5.6 ± 3.0; C: 4.9 ± 3.7 (mothers)  I_1_: 3.2 ± 2.3; C: 2.6 ± 2.3 (fathers) | I_1_: 2.0 ± 1.7; C: 3.8 ± 2.4 (mothers)  I_1_: 0.8 ± 1.0; C: 1.7 ± 1.6 (fathers) | -2.1 [95% CI: -3.1;-1.2] (mothers)  -1.1 [95% CI: -1.8;-0.4] (fathers) |  |
| Feeley et al.^15^ | I_1_: 5.5 ± 2.9; C: 5.5 ± 2.9 | I_1_: 2.4 ± 2.9; C: 3.2 ± 3.3 |  |  |
| Holditch-Davis et al.^16,a^ |  |  |  | I_1_: -0.17 (SE 0.45), time x I_1_: 0.006 (SE 0.008); I_2_: 0.05 (SE 0.45), time x I_2_: 0.008 (SE 0.009); time: -0.08 (SE 0.01), time squared: 0.001 (SE 0.000) |
| Horsch et al.^17^ | I_1_: 4.09 ± 2.9; C: 4.14 ± 3.2 | I_1_: 2.60 ± 2.50; C: 3.64 ± 3.10 (1^st^ assessment)  I_1_: 2.78 ± 2.70; C: 4.05 ± 3.30 (2^nd^ assessment) |  |  |
| Izadi et al.^18^ | I_1_: 45.16 ± 8.80; C: 44.00 ± 7.92 | I_1_: 32.73 ± 9.79; C: 42.33 ± 9.92 (1^st^ assessment)  I_1_: 31.53 ± 9.59; C: 37.40 ± 10.02 (2^nd^ assessment) |  |  |
| Koochaki et al.^19^ | I_1_: 8.095 ± 5.202; C: 6.820 ± 4.297 | I_1_: 4.547 ± 2.297; C: 6.564 ± 4.290 (1^st^ assessment)  I_1_: 5.000 ± 2.447; C: 14.205 ± 4.856 (2^nd^ assessment) |  |  |
| Pourmovahed et al.^20^ | I_1_: 9.39 ± 1.67; C: 8.54 ± 1.59 | I_1_: 4.39 ± 1.49; C: 5.31 ± 1.17 | -0.92 ± 0.41 |  |
| Shaw et al.^21^ | I_1_: 29.25 ± 11.52 | I_1_: 27.13 ± 13.94 | -2.13 |  |
| Shaw et al.^10^ | I_1_: 49.40 ± 25.49; C: 42.35 ± 27.05 |  |  | I_1_: -12.886 (95% CI: -17.143;-8.359); C: -5.509 (95% CI: -10.732;-0.285) → group difference: -7.378 (95% CI: -13.724;1.032) |
| Shaw et al.^22^ | I_1_: 49.40 ± 25.49; C: 42.35 ± 27.05 |  |  | I_1_: -7.347 (95% CI: -14.394;-0.301) (1^st^ assessment)  I_1_: -15.996 (95% CI: -23.128;-8.863) (2^nd^ assessment) |
| Simon et al.^23^ | I_1_: 47.32 ± 30.41 | I_1_: 41.92 ± 26.67 (1^st^ assessment)  I_1_: 24.00 ± 16.33 (2^nd^ assessment) |  |  |
| Zelkowitz et al.^24^ | I_1_: 5.7 ± 3.1; C: 5.6 ± 2.9 | I_1_: 2.9 ± 2.9; C: 3.2 ± 2.8 | I_1_: -2.6 ± 2.9; C: -2.2 ± 3.3; 95% CI: -0.8;1.5 |  |

Abbreviations: C, control group; CI, confidence interval; I_1_, intervention group n.1; I_2_, intervention group n.2; PTSD, post-traumatic stress disorder; SD, standard deviation; SE, standard error

^§^According to mean (± SD) PTSD score at baseline, most studies included both patients with PTSD score above the cut-off for diagnosis of PTSD and patients with PTSD score under the clinical range^10-15,17,18,21-24^. In the studies by Koochaki et al. and Pourmovahed et al., only patients with PTSD score in clinical range were included^19,20^. In the study by Holditch-Davis et al., the percentage of patients with PTSD score in clinical range was not specified^16^; *mean (SE); °after 1^st^ stage of the intervention

^a^General linear models indicated that the study groups did not differ for PTSD at enrollment (data not shown). Time and time squared effects indicated that post-traumatic stress symptoms decreased over time and the rate of decrease slowed over time.
