# Supplementary material for: Symptoms of post-traumatic stress disorder in parents of preterm newborns: A systematic review of interventions and prevention strategies
Source: Front Psychiatry. 2023 Mar 8;14:998995. doi: 10.3389/fpsyt.2023.998995 (PMC10032332; doi:10.3389/fpsyt.2023.998995)
Supplement: Supplementary file 6 [file Table_6.DOCX]

Table 6. Effects of interventions on PTSD development in the studies included in our systematic review. Details about interventions administered to each study group are given in Table 4.

|  | Barlow et al.^11^ | Bernard et al.^12^ | Borghini et al.^13,a^ | Castel et al.^14^ | Feeley et al.^15^ | Holditch-Davis et al.^16,b^ | Horsch et al.^17,a^ | Izadi et al.^18,a^ | Koochaki et al.^19,a^ | Pourmovahed et al.^20^ | Shaw et al.^21^ | Shaw et al.^10^ | Shaw et al.^22,a^ | Simon et al.^23,a^ | Zelkowitz et al.^24^ |
| --- | --- | --- | --- | --- | --- | --- | --- | --- | --- | --- | --- | --- | --- | --- | --- |
| Intervention group n.1 | n.a. | n.a. | ↓ | n.a. | n.a. | n.s. | ↓ | ↓ | ↓ | ↓ | n.s. | ↓ | ↓ | ↓ | ↓ |
| Intervention group n.2 |  |  |  |  |  | n.s. |  |  |  |  |  |  | ↓ |  |  |
| Control group | n.a. | n.a. | n.a. | n.a. | n.a. | n.a. | n.s. | ↓ | n.a. | n.a. |  | ↓ | n.a. |  | ↓ |
| Intervention group n.1 vs Intervention group n.2 |  |  |  |  |  | n.s. |  |  |  |  |  |  | n.s. |  |  |
| Intervention group n.1 vs Control group | n.s. | n.s. | n.s. | I_1_ | n.s. | n.s. | n.s. | I_1_ | I_1_ | I_1_ |  | I_1_ | I_1_ |  | n.s. |
| Intervention group n.2 vs Control group |  |  |  |  |  | n.s. |  |  |  |  |  |  | I_2_ |  |  |

Legend: n.a., not available (data not shown or statistical significance of data not demonstrated); n.s., not significant; ↓, significant decrease in PTSD symptoms; I_1_, lower post-traumatic stress symptoms in intervention group n.1; I_2_, lower post-traumatic stress symptoms in intervention group n.2

^a^In case of multiple PTSD assessments after the end of an intervention, the most recent one has been taken into account for the purposes of this systematic review; ^b^As regards the longitudinal effects of the interventions, KC, ATVV, and control mothers did not differ on post-traumatic stress symptoms (data not shown)
