# Supplementary material for: Symptoms of post-traumatic stress disorder in parents of preterm newborns: A systematic review of interventions and prevention strategies
Source: Front Psychiatry. 2023 Mar 8;14:998995. doi: 10.3389/fpsyt.2023.998995 (PMC10032332; doi:10.3389/fpsyt.2023.998995)
Supplement: Supplementary file 7 [file Table_7.DOCX]

Table 7. Risk of bias assessment for included randomized controlled trials^36^

|  | Barlow et al^11^ | Bernard et al^12^ | Borghini et al^13^ | Castel et al^14^ | Feeley et al^15^ | Holditch-Davis et al^16^ | Horsch et al^17^ | Izadi et al^18^ | Koochaki et al^19^ | Pourmovahed et al^20^ | Shaw et al^10^ | Shaw et al^22^ | Zelkowitz et al^24^ |
| --- | --- | --- | --- | --- | --- | --- | --- | --- | --- | --- | --- | --- | --- |
| R | Low | Low | Some concerns | Low | Low | Low | Low | Some concerns | Some concerns | Some concerns | Low | Low | Low |
| D | Low | Some concerns | Low | High | Low | Low | Low | Low | Low | Low | Low | High | Low |
| Mi | Low | Low | Low | Low | Low | Low | Low | Low | Low | Low | Low | Low | Low |
| Me | Low | Low | Low | Low | Low | Low | Low | Low | Low | Low | Low | Low | Low |
| S | Low | Low | Low | Low | Low | Low | Low | Low | Low | Low | Low | Low | Low |
| O | Low | Some concerns | Some concerns | High | Low | Low | Low | Some concerns | Some concerns | Some concerns | Low | High | Low |

Abbreviations: D, bias due to deviations from intended interventions; Me, bias in measurement of the outcome; Mi, bias due to missing outcome data; O, overall risk of bias; R, bias arising from the randomisation process; S, bias in selection of the reported result.
