# Supplementary material for: Symptoms of post-traumatic stress disorder in parents of preterm newborns: A systematic review of interventions and prevention strategies
Source: Front Psychiatry. 2023 Mar 8;14:998995. doi: 10.3389/fpsyt.2023.998995 (PMC10032332; doi:10.3389/fpsyt.2023.998995)
Supplement: Supplementary file 8 [file Table_8.DOCX]

Table 8. Risk of bias assessment for included before-after studies^37^

|  | | Shaw et al^21^ | Simon et al^23^ |
| --- | --- | --- | --- |
| Questions | Was the study question or objective clearly stated? | Yes | Yes |
|  | Were eligibility/selection criteria for the study population prespecified and clearly described? | Yes | Yes |
|  | Were the participants in the study representative of those who would be eligible for the test/service/intervention in the general or clinical population of interest? | Yes | Yes |
|  | Were all eligible participants that met the prespecified entry criteria enrolled? | Cannot determine | Cannot determine |
|  | Was the sample size sufficiently large to provide confidence in the findings? | No | No |
|  | Was the test/service/intervention clearly described and delivered consistently across the study population? | Yes | Yes |
|  | Were the outcome measures prescpecified, clearly defined, valid, reliable, and assessed consistently across all study participants? | Yes | Yes |
|  | Were the people assessing the outcomes blinded to the participants’ exposures/interventions? | Not applicable | Not applicable |
|  | Was the loss to follow-up after baseline 20% or less? Were those lost to follow-up accounted for in the analysis? | No | No |
|  | Did the statistical methods examine changes in outcome measures from before to after the intervention? Were statistical tests done that provided p values for the pre-to-post changes? | Yes | Yes |
|  | Were outcome measures of interest taken multiple times before the intervention and multiple times after the intervention (i.e., did they use an interrupted time-series design?) | No | No |
|  | If the intervention was conducted at a group level (e.g., a whole hospital, a community, etc.) did the statistical analysis take into account the use of individual-level data to determine effects at the group level? | Not applicable | Not applicable |
|  | Quality rating | Poor | Poor |
